# Imbalanced LIMK1 and LIMK2 expression leads to human colorectal cancer progression and metastasis via promoting β-catenin nuclear translocation

**DOI:** 10.1038/s41419-018-0766-8

**Published:** 2018-07-03

**Authors:** Yue Zhang, Aimin Li, Jiaolong Shi, Yuxin Fang, Chuncai Gu, Jianqun Cai, Chuang Lin, Liang Zhao, Side Liu

**Affiliations:** 1 Guangdong Provincial Key Laboratory of Gastroenterology, Department of Gastroenterology, Nanfang Hospital, Southern Medical University, Guangzhou, Guangdong China; 2 Department of General Surgery, Nanfang Hospital, Southern Medical University, Guangdong Provincial Engineering Technology Research Center of Minimally Invasive Surgery, Guangzhou, Guandong China; 3 Department of pathology, Nanfang Hospital, Southern Medical University, Guangzhou, Guandong China

## Abstract

Epithelial–mesenchymal transition (EMT)-induced metastasis contributes to human colorectal cancer (CRC) progression, especially in advanced CRC. However, the underlying mechanism of β-catenin in this process is elusive. We identified that LIM domain kinase (LIMK)2 was progressively downregulated with tumor progression from precancerous lesions to advanced cancer. Gain- and loss-of-function assays revealed that LIMK2 inhibits cell proliferation via cell cycle arrest at the G1–S transition and suppresses the ability of cell metastasis by restricting the EMT process. Reduced LIMK2 expression enhanced the nuclear accumulation of β-catenin and activated the Wnt signaling pathway, thus contributing to tumor progression. A homolog of the LIMK family, LIMK1, which was overexpressed throughout tumor progression, served as a competitive inhibitor of LIMK2 via β-catenin nuclear translocation. The imbalanced expression of LIMK1 and LIMK2 is important in CRC progression, and the combined effects provide a new insight into the mechanism of CRC progression. These findings provide a new understanding for LIMK-based anticancer therapy.

## Introduction

Colorectal cancer (CRC) is one of the most common cancers and the leading causes of cancer death in China^1^, with a growth rate that is twice that of the world average^2^. CRC is a heterogeneous disease and usually occurs from precancerous lesions^3^. Serrated adenoma (SA) is a type of adenoma with serrated crypt structure, including hyperplastic polyps, sessile-serrated adenoma/polyp (SSA/P) and traditional serrated adenoma. SA is a kind of precancerous lesion and has high incidence of CRC, especially the SSA/P subtype^4^. The saw-tooth-like epithelium has been considered as a consequence of expanding the crypt proliferation zone and inhibiting programmed cellular exfoliation^5^. Activation of the Wnt signaling pathway contributes to the malignant transformation of SA^6^. By improving our understanding of the process of SA transitioning into CRC, we may propose an effective marker for tumor progression. For CRC patients, metastasis is a noteworthy cause of lethality. CRC metastasis is a multi-step, multi-stage, polygene process^7^, and the molecular framework that involves tumor metastasis has been found in recent years^8^. However, there are limited effective biomarkers for early tumor metastasis. Hence, it is still important to uncover the molecular mechanism underlying CRC metastasis.

LIM domain kinase (LIMK) is a serine–threonine kinase that contains two LIM motifs at the N-terminus and a kinase sequence at the C-terminus^9^. The LIM domain can combine with proteins, and the kinase can phosphorylate downstream genes. Two distinct protein kinases belong to the LIMK family: LIMK1 and LIMK2. Previous studies have identified LIMK1 as a cancer-promoting regulator in multiple organ cancers, such as breast cancer^10^, prostate cancer^11^, and CRC^12^. Though LIMK2 has an overall sequence similar to LIMK1, LIMK2 has specific functions in different organs. Increased LIMK2-induced drug-resistance has been observed in neuroblastoma^13^. Phosphorylation of LIMK2 is pivotal for breast cancer progression and metastasis^14^. In CRC, LIMK2 has been shown to limit stem cell proliferation^15^. However, the mechanism of LIMK2 in CRC progression and metastasis is still not clear. Interestingly, our previous study found that LIMK2 displays an opposite expression level to that of LIMK1 in CRC^12^. The correlation between LIMK1 and LIMK2 has not been characterized before. Therefore, we suppose that LIMK2 may serve a different role than LIMK1 in CRC development.

In this study, we found that the expression of LIMK2 was progressively reduced from normal tissues to precancerous lesions (SAs) to CRC tissues. Silencing LIMK2 remarkably promoted CRC formation and metastasis in vitro and in vivo through activating the Wnt/β-catenin signaling pathway. Furthermore, we identified that LIMK2 expression was negatively correlated with that of LIMK1. The overexpression of LIMK1 and reduction in LIMK2 led to combined effect of β-catenin nuclear accumulation. These findings may provide new insight into LIMK-based target therapy for CRC.

## Results

### LIMK2 is progressively downregulated in human CRC tissues

**Fig. 1:**
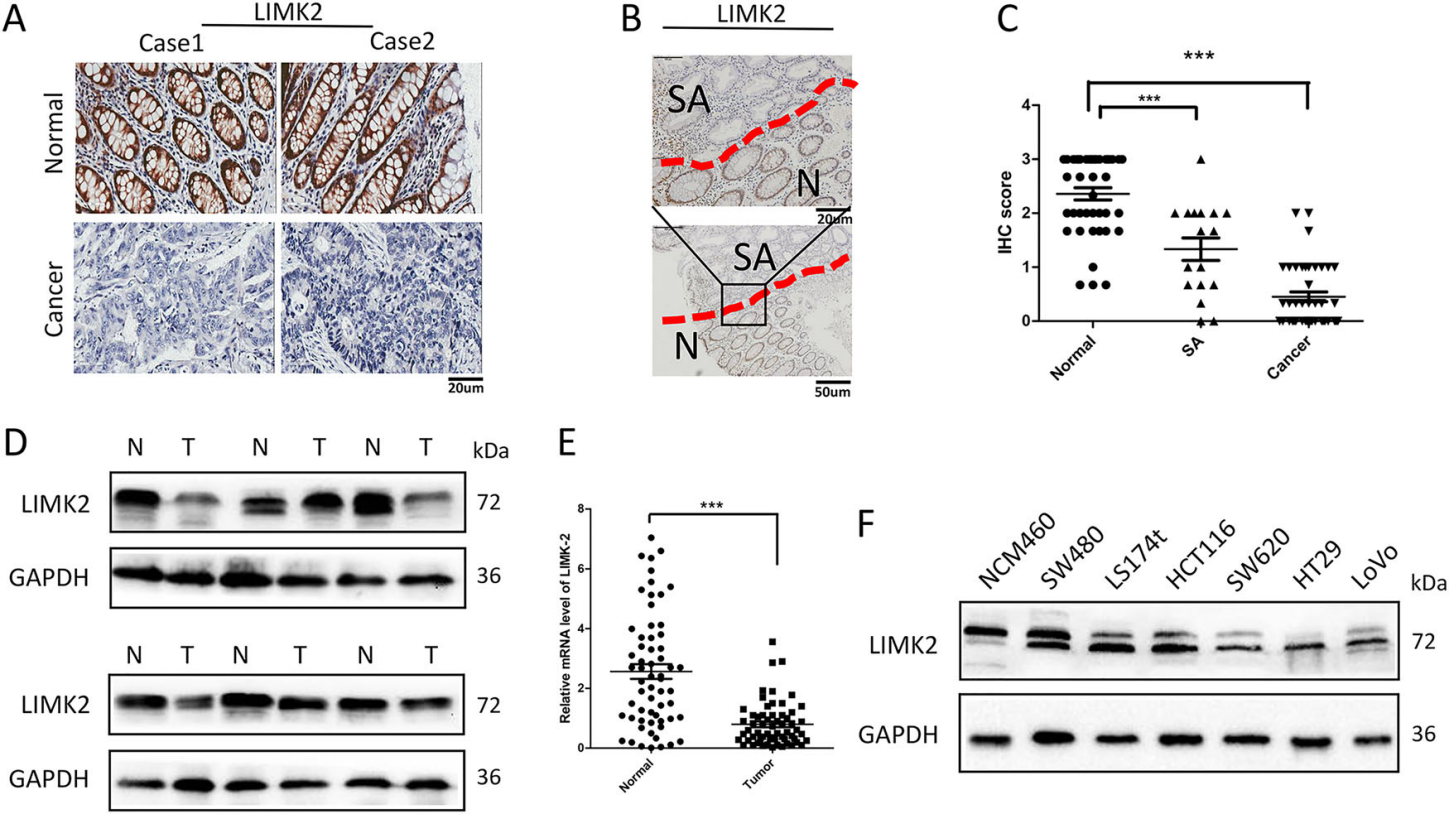
LIMK2 is progressively downregulated in human CRC tissues. **a** IHC analysis of LIMK2 protein expression in 42 paired CRC tissues and adjacent non-tumor tissues (15 from a TMA, 27 from surgery). Representative photographs are shown. **b** IHC analysis of LIMK2 protein expression in 17 SA tissues and adjacent normal mucosa (the upper panel, magnification, × 400; scale bar: 20 µm; the lower panel, magnification, × 200; scale bar: 50 µm). **c** IHC scores of normal mucosa, SA and CRC tissues. **d** Western blot analysis of LIMK2 in 16 paired tumor tissues T and adjacent non-tumor tissues N. Representative photographs are shown. **e** Real-time PCR analysis of LIMK2 expression of 65 paired human CRC tissues and their adjacent normal mucosa tissues. **f** LIMK2 expression was detected by western blotting in CRC cell lines

SA is a type of precancerous lesion to CRC, and 15–30% of CRC diagnoses occur from SAs; thus, SA presents a favorable model to study carcinogenesis. The expression of LIMK2 in 15 paired CRC tissues and normal colorectal mucosa tissues from a tissue microarray, 27 paired CRC tissues and normal colorectal mucosa tissues from patients who had undergone surgical resection, and 17 SA tissues was detected via immunohistochemistry (IHC). LIMK2 was downregulated in CRC tissues compared with the LIMK2 expression in the adjacent normal tissues (Fig. 1a). Compared with the normal colon epithelial tissues, decreased expression of LIMK2 was frequently observed in the SA tissues (Fig. 1b). The exactly IHC score was attached as Supplementary Table 1. LIMK2 was reduced in SA and CRC tissues to various degrees, and LIMK2 expression was progressively downregulated with the advancement of tumor development (Fig. 1c). We detected that LIMK2 expression was decreased in 13 of 16 CRC tissues compared with that in the paired normal colon mucosa by western blot (WB; Fig. 1d; Supplementary S1A). Real-time PCR analysis verified that LIMK2 was downregulated in 65 human CRC tissues and their adjacent normal mucosa tissues (Fig. 1e). LIMK2 expression varies in different colorectal cell lines, such as NCM460, SW480, LS174t, HCT116, SW620, HT29, and LoVo (Fig. 1f). Compared with the relatively low metastatic potential cell lines, such as SW480 and HCT116, LIMK2 was significantly reduced in the LoVo and SW620 cell lines with a relatively high metastatic potential. These results indicated that LIMK2 might be involved in CRC progression.

### Reduced LIMK2 contributes to aggressive phenotypes of cell lines in vitro

**Fig. 2:**
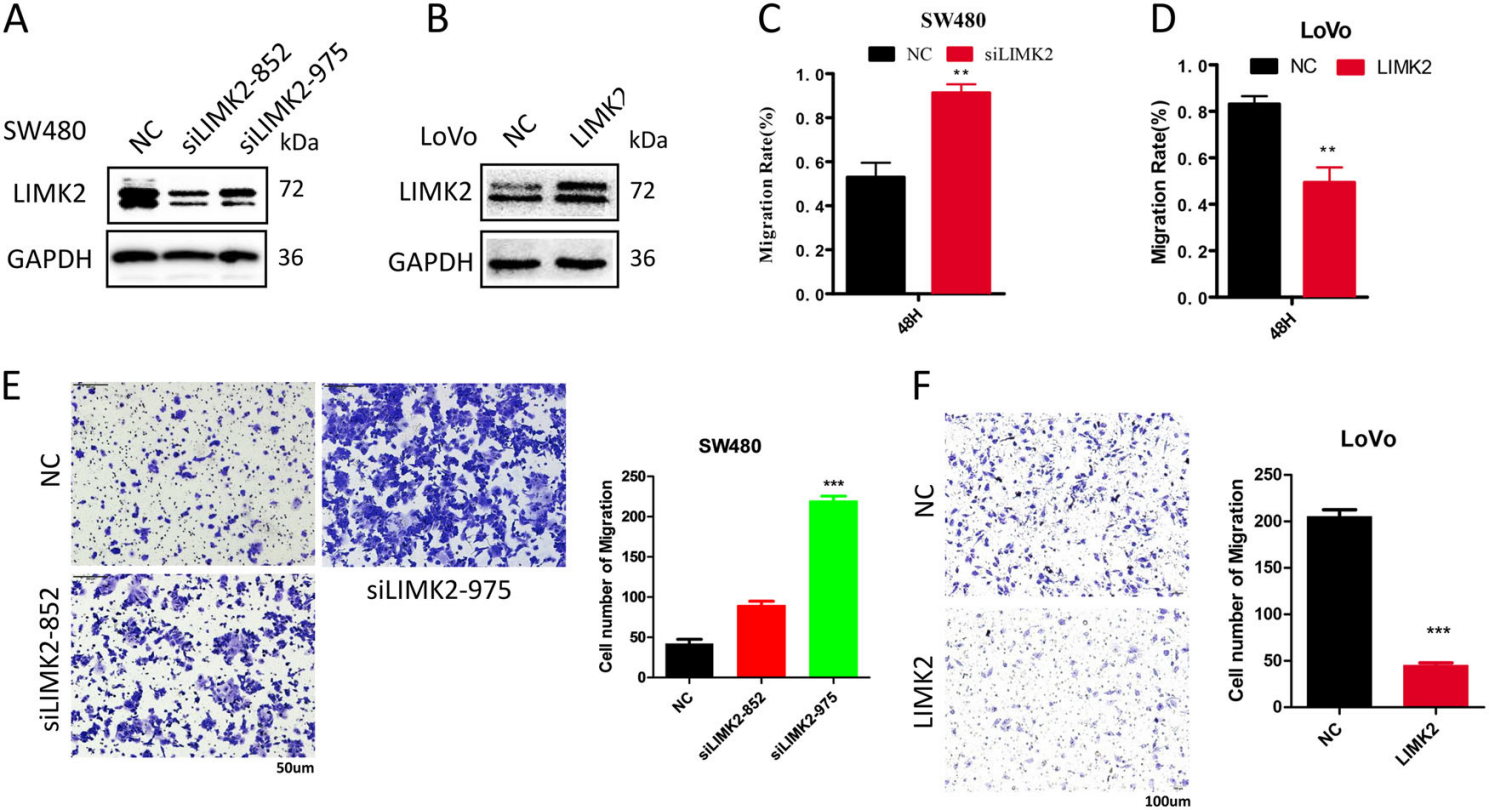
Reduced LIMK2 contributes to aggressive phenotypes of cell lines in vitro. **a**, **b** SW480 cells were transiently transfected with siLIMK2 and siRNA NC. LoVo cells were transiently transfected with LIMK2 and NC vectors. Western blot analysis was performed to detect the expression of LIMK2. **c**, **d** Wound-healing assays were used for detecting the migration ability in LIMK2 knocked down or overexpression CRC cell lines. Bars represent migration index of treated or control cells. The distance migrated by treated cells was relative to that migrated by control cells. Representative figures are shown. Error bars represent mean ± S.D. from three independent experiments. The asterisk (*) indicates *P*<0.05. The double asterisk (**) indicates *P*<0.01. **e**, **f** The invading cells of the transwell assay were counted under a microscope in five randomly selected fields. Bars represent the number of invaded cells. Error bars represent mean ± S.D. from three independent experiments. ***P<0.001

We found that LIMK2 was involved in colorectal progression at the tissue level. To evaluate the biological function of LIMK2 in CRC cell lines, we used siRNA-mediated RNA interference to carry out an in vitro loss-of-function analysis in SW480 and HCT116 cells. Two siRNAs (siRNA-852 and siRNA-975) were constructed for further examination. For the in vitro gain-of-function analysis, a plasmid was used to transfect LoVo and HT29 cells. WB confirmed the transfection efficiency in both cell lines (Fig. 2a, b). Wound healing and transwell assays revealed that knocking down LIMK2 increased the migration and invasion abilities of CRC cells (Fig. 2c, e). Conversely, overexpression of LIMK2 achieved the opposite effect (Fig. 2d, f). We have attached the representative images of wound healing for each time point and conditions in Supplementary S1B. We also achieved the same results in silencing LIMK2 in HCT116 cells and overexpression of LIMK2 in HT29 cells (supplementary S2A–S2D). Using flow cytometry, we investigated the role of LIMK2 on cell cycle regulation using propidium iodide staining. Silencing LIMK2 accelerated the G1–S phase transition, whereas LIMK2 overexpression arrested the G1–S phase transition (supplementary S3A). We assessed the cell cycle protein using WB, the results were shown in Supplementary S3A. Next, we conducted CCK8 to assess LIMK2 on cell proliferation (Supplementary S3B). Decreased LIMK2 accelerated G1–S phase transition, and the cell cycle regulation genes CyclinD1, CDK4 increased the expression level, and cell proliferation ability was enhanced. Increased LIMK2 arrested G1–S phase transition, CyclinD1, CDK4 were downregulated, and the cell proliferation ability was suppressed. These results revealed that LIMK2 contributes to CRC cell metastasis and cell cycle regulation.

### Knocking down LIMK2 promotes CRC progression and metastasis in vivo

**Fig. 3:**
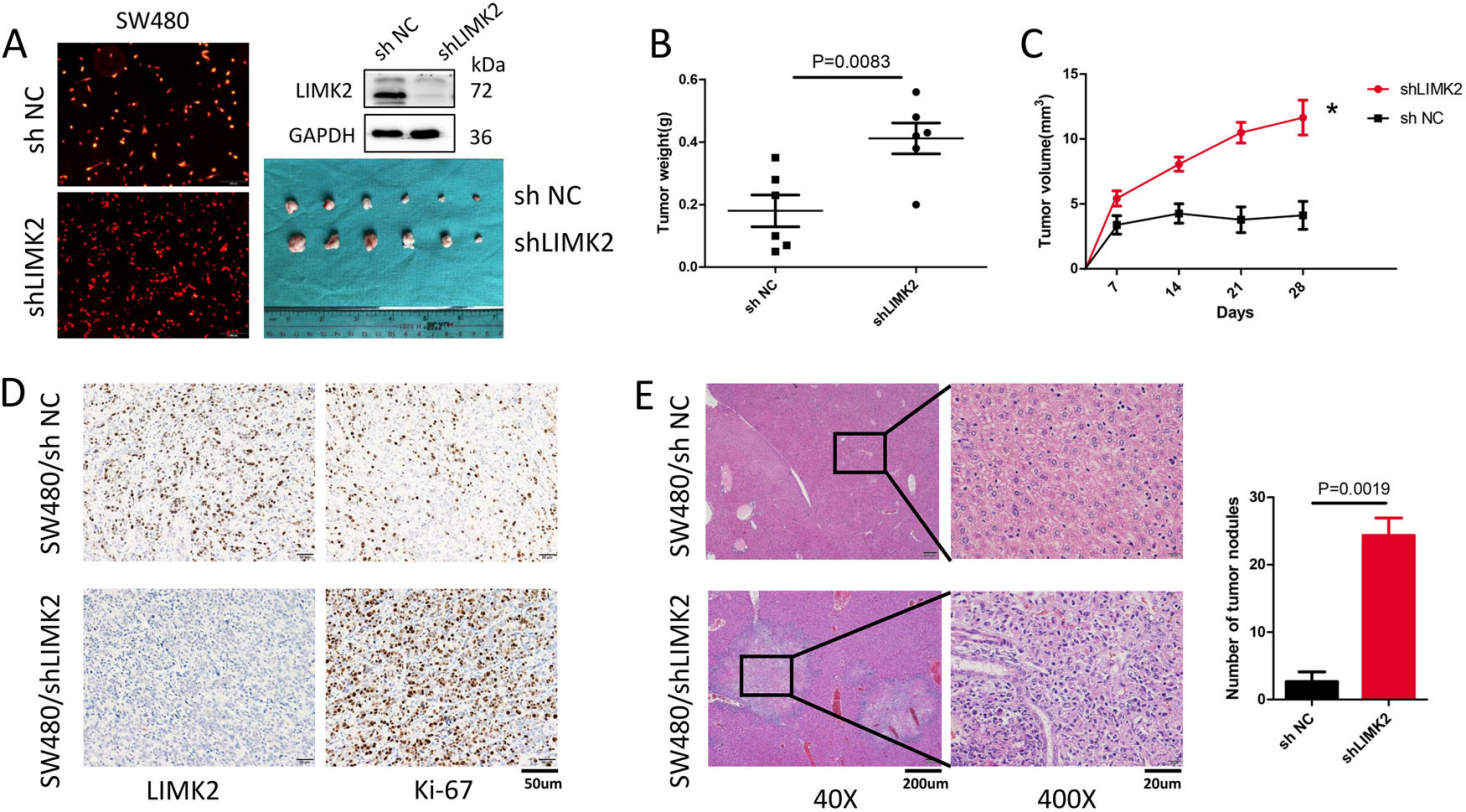
Knocking down LIMK2 promotes CRC progression and metastasis in vivo. **a** LIMK2 shRNA-transduced stable SW480 cells were injected subcutaneously into the back of nude mice to evaluate tumor growth (*n* = 6). Representative figure of tumors formed is shown. **b**, **c** Tumor weight and volume in the back of nude mice injected with indicated cells was measured. The data of all primary tumors are expressed as mean ± S.D. Scatter plots of tumor weight derived from indicated cells at 30 d after subcutaneous implantation. **d** The representative photographs of LIMK2 staining of subcutaneous tumor are shown (magnification, × 200; scale bar: 50 µm). Proliferative ability was indicated by the Ki-67. **e** Tumor cells were injected into nude mice through the spleen to evaluate the liver homing capacity of cells (*n* = 6), respectively (the left panel, magnification, × 40; scale bar: 200 µm; the right panel, magnification, × 400; scale bar: 20 µm). The number of metastatic liver nodules in individual mice was counted under the microscope and expressed as mean ± S.D

We revealed that LIMK2 promotes an aggressive phenotype in CRC cells in vitro. To identify the effect of LIMK2 on CRC genesis and metastasis in vivo, we established stable LIMK2-silencing cell lines using a specific shRNA in SW480 cells. As shown in Fig. 3a, the transfected cells were shown with red fluorescence, and WB verified the transfection efficiency. First, a subcutaneous tumor model was established to evaluate the tumorigenesis of LIMK2. The tumors from SW480/shLIMK2 xenografts grew faster and larger than those in the control group (Fig. 3b, c). Furthermore, the LIMK-silenced group showed a higher Ki-67 percentage than the control group (Fig. 3d).

To assess the effect of LIMK2 on tumor-homing capacity in vivo, nude mice (*n* = 6, male) were injected with the constructed cells through their spleen. Compared with the control group, more and larger tumor nodules were found in the livers of mice in the LIMK2-silenced group (Fig. 3e). These data strongly suggested that LIMK2 promotes tumor progression and metastasis capabilities in vivo.

### Knocking down LIMK2 promotes β-catenin nuclear translocation and activates the Wnt/β-catenin signaling pathway

**Fig. 4:**
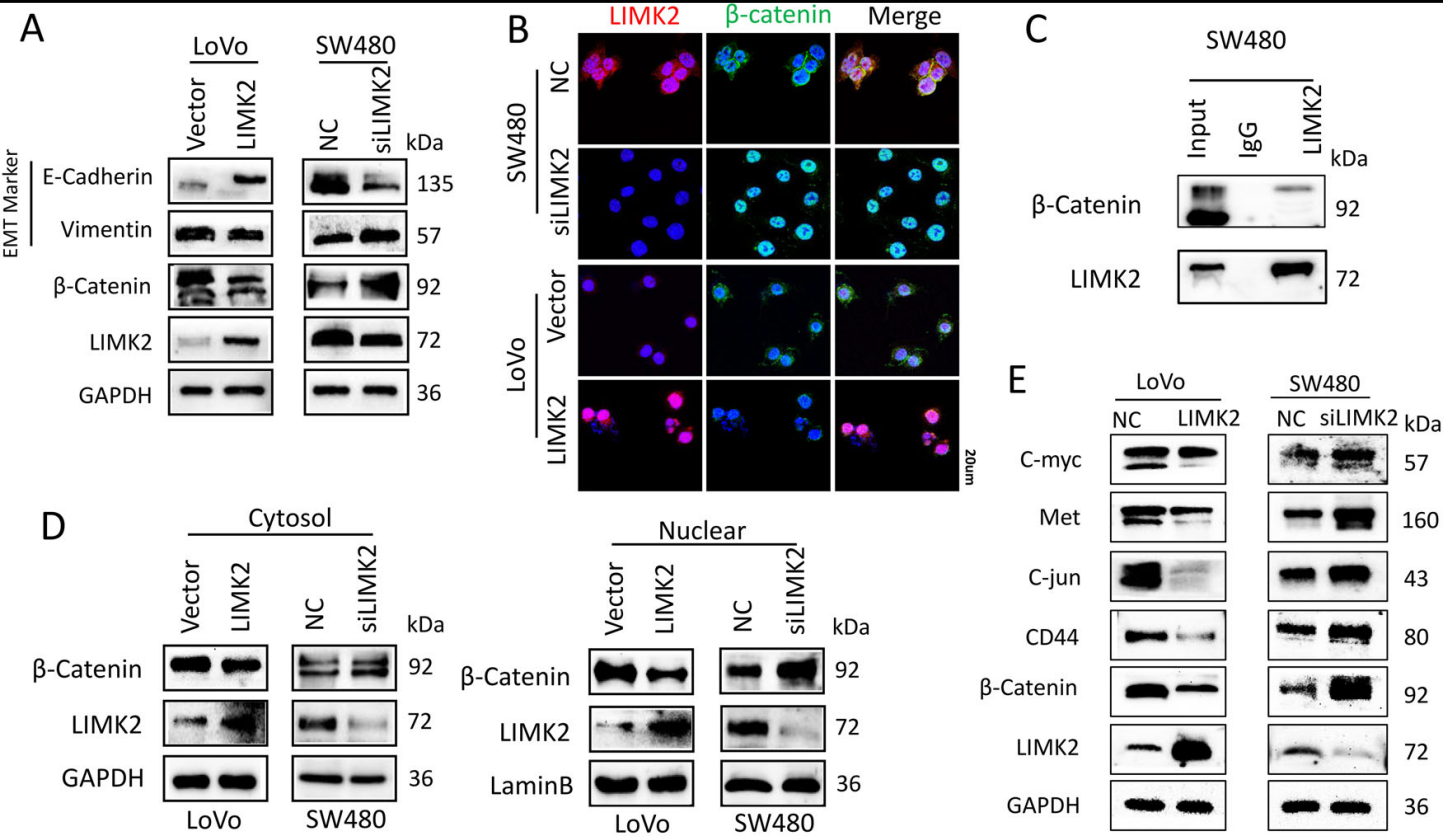
Knocking down LIMK2 promotes β-catenin nuclear translocation and activates the Wnt/β-catenin signaling pathway. **a** Western blot analysis of EMT markers (E-cadherin, vimentin) in indicated cells treated with siLIMK2 and LIMK2 vector. **b** Immunofluorescence assays of β-catenin in the treated cells, as indicated. Representative merge figures are shown (magnification, × 400; scale bar: 20 µm). **c** Co-immunoprecipitation revealed the relationship between LIMK2 and β-catenin in SW480 cells. **d** Western blot analysis of cytoplasmic and nuclear fractions from SW480 and LoVo. Nuclear segregation is assayed by total Lamin B. Cytoplasmic segregation is assayed by GAPDH. **e** Expression level of targeted genes activated by Wnt signaling pathways were detected in SW480 and LoVo

To further understand the mechanism of the LIMK2-mediated aggressive phenotype in colorectal cells, we performed WB to detect the expression of markers associated with the epithelial–mesenchymal transition (EMT). WB showed that the mesenchymal marker (vimentin) was upregulated, whereas the epithelial marker (E-cadherin) was downregulated after knocking down LIMK2. However, β-catenin showed the opposite effect (Fig. 4a). As shown in Fig. 4b, knocking down LIMK2promoted β-catenin nuclear translocation. Co-immunoprecipitation validated that LIMK2 directly interacts with β-catenin in SW480 cells (Fig. 4c). WB showed that the abundance of β-catenin was upregulated both in the cytoplasm and nucleus with the LIMK2 knockdown (Fig. 4d). We hypothesized that loss of LIMK2 activated the Wnt/β-catenin signaling pathway by regulating the nuclear translocation of β-catenin. WB showed that the decrease in LIMK2 expression activated the downstream target genes of the Wnt/β-catenin signaling pathway (Fig. 4e). These data indicated that downregulation of LIMK2 activated the Wnt signaling pathway through promoting β-catenin nuclear translocation.

### Imbalance of LIMK1/LIMK2 promotes CRC aggression via regulating the Wnt/β-catenin signaling pathway

**Fig. 5:**
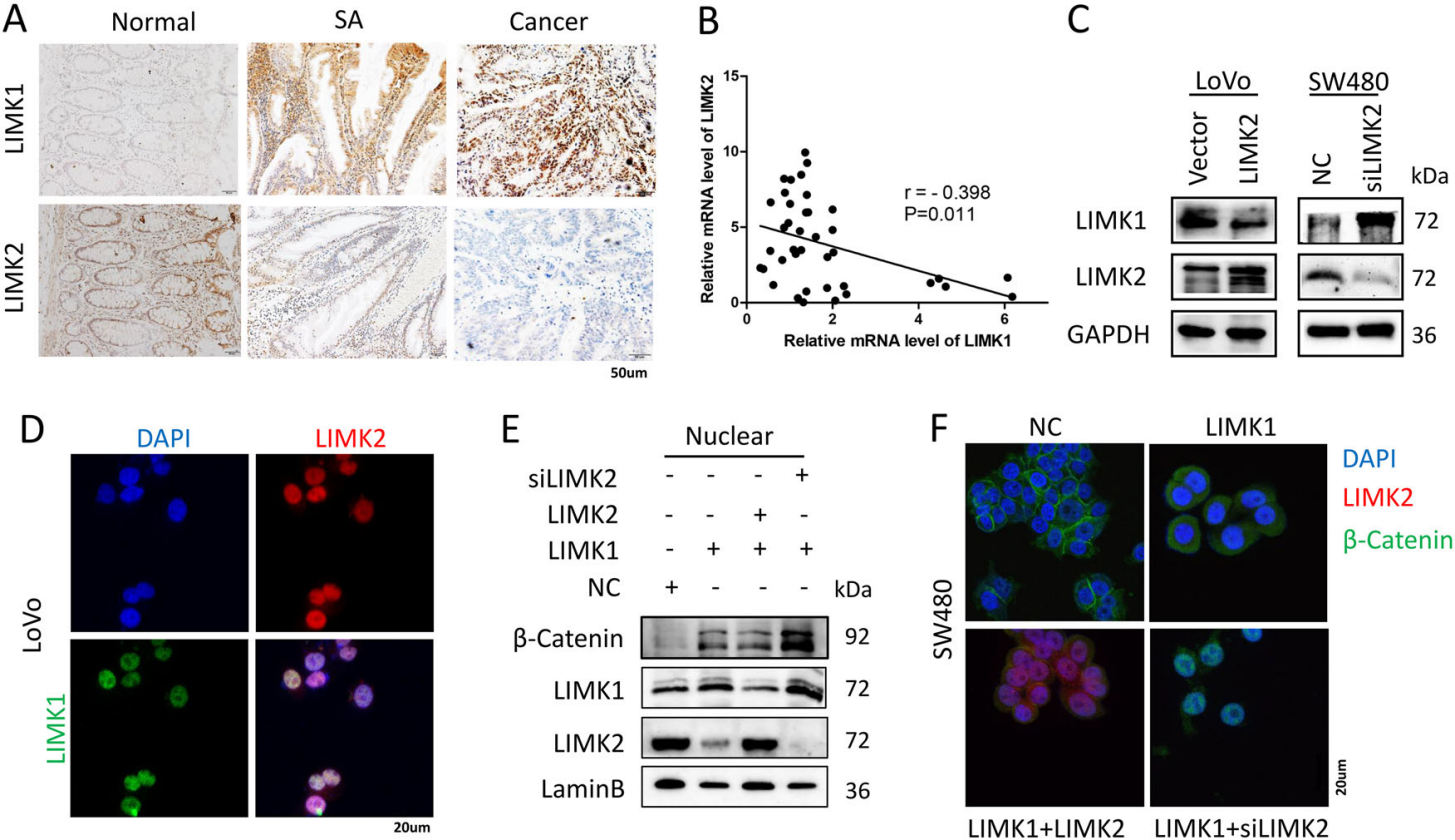
Imbalance of LIMK1/LIMK2 promotes β-catenin nuclear accumulation . **a** IHC staining of LIMK1, LIMK2 in 27 normal and CRC tissues,17 SA tissues. Representative figures are shown (magnification, × 200; scale bar: 50 µm). **b** Real-time PCR analysis of LIMK1 and LIMK2 in 40 normal mucosa tissues. Correlation analysis were shown by Spearman’s correlation analyses between LIMK1 expression and LIMK2. **c** Expression of LIMK1 was detected by WB in CRC cell lines with LIMK2 knocked down or overexpressed, respectively. **d** IF analysis for subcellular localization of LIMK1-HA and LIMK2-Flag in LoVo cells (magnification, × 400; scale bar: 20 µm). **e** Nuclear proteins from SW480 and SW480 transfected with LIMK1, transfected with LIMK2 or siLIMK2, respectively, were used for detecting the indicated proteins of the LIMK1, LIMK2 and β-catenin by WB. **f** Immunofluorescence assays of LIMK2 and β-catenin proteins in SW480-transduced cells, as indicated. Representative merge figures are shown (magnification, × 400; scale bar: 20 µm)

**Fig. 6:**
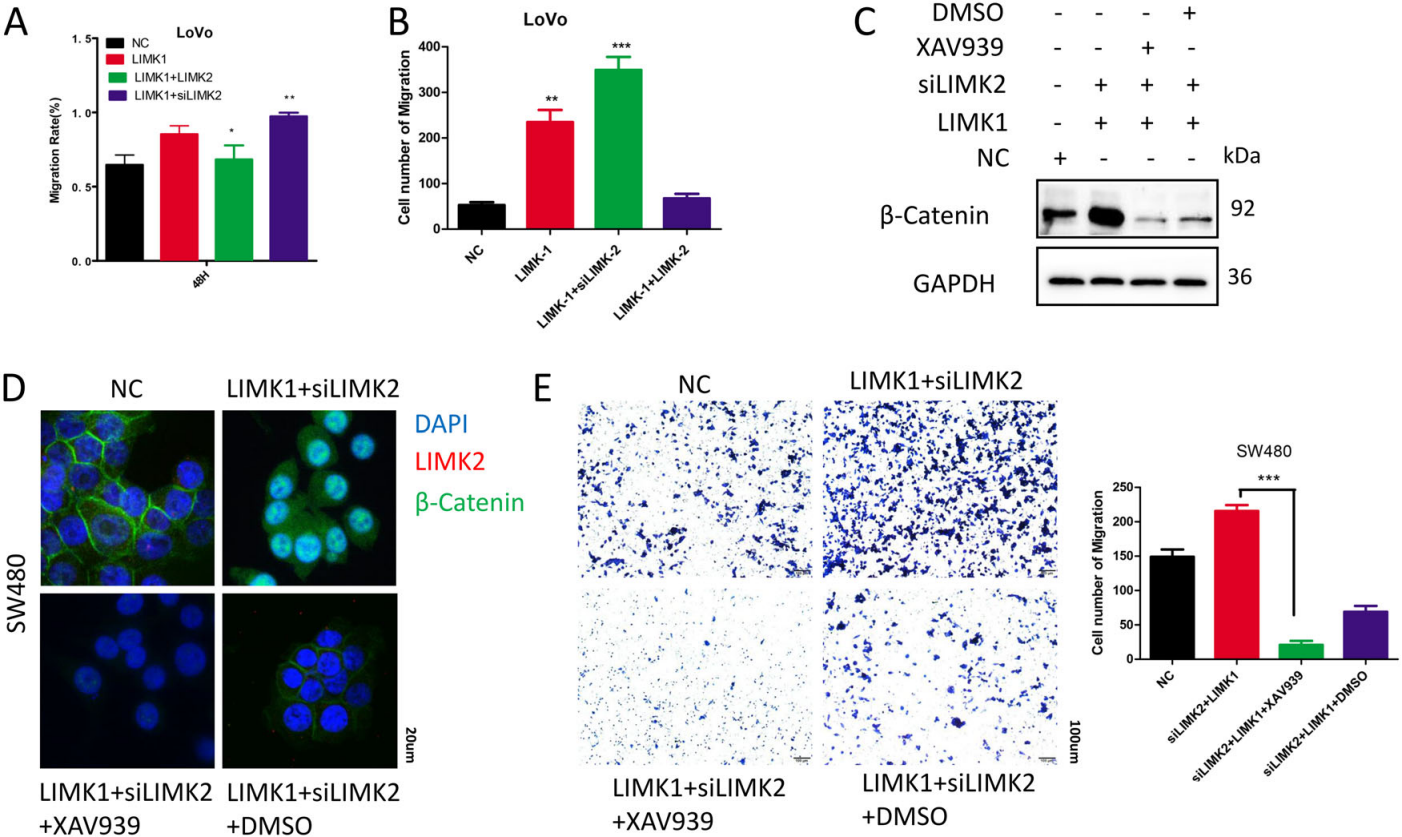
Imbalance of LIMK1/LIMK2 promotes CRC aggression via regulating WNT/β-catenin signaling pathway. **a** Wound-healing assay of cells overexpressing LIMK1 and co-transfected with siLIMK2 or LIMK2 plasmid, as indicated. **b** Transwell assay of cells overexpressing LIMK1 and co-transfected with siLIMK2 or LIMK2 plasmid, as indicated. The cells were counted under a microscope in five randomly selected fields. Bars represent the number of invaded cells. **c** Whole proteins from LIMK2 knocked down cell lines, co-transfected with LIMK1 vector cells, and co-transfected cells treated with the β-catenin inhibitorXAV939 (5 μM) or DMSO for 24 h, were used for detecting the indicated proteins of the Wnt/β-catenin pathway by WB. **d** IF assays verified β-catenin abundance in LIMK2 knocked down cell lines, co-transfected with LIMK1 vector cells, and co-transfected cells treated with XAV939 (5 μM) or DMSO for 24 h. Representative merge figures are shown (magnification, × 400; scale bar: 20 µm). **e** LIMK2 knocked down cell lines, co-transfected cells, and co-transfected cells treated with the XAV939 (5 μM) were measured migration ability by Transwell assays. Error bars represent mean ± S.D. from three independent experiments. ***P<0.001

In our previous study, we found an interesting phenomenon that the expression of LIMK2 was opposite to the expression of LIMK1^12^. In normal mucosa, SA, and CRC tissues, we detected LIMK1 and LIMK2 expression levels individually by IHC (Fig. 5a). In normal tissues, LIMK1 showed low expression, whereas LIMK2 showed high expression. During CRC genesis, the balance of LIMK1/LIMK2 seemed to be reversed; LIMK1 was overexpressed, whereas LIMK2 was progressively reduced. RT-PCR also validated the negative correlation between LIMK1 and LIMK2 in normal colon mucosa (Fig. 5b). To investigate whether LIMK2 affected the expression of LIMK1, we performed WB (Fig. 5c), and LIMK2 knockdown increased LIMK1 expression. Immunofluorescence assays revealed the colocalization of LIMK1 and LIMK2 (Fig. 5d). A previous study showed that LIMK1 was correlated with β-catenin^16^. Immunoprecipitation validated that LIMK1 interacts with β-catenin (supplementary S4A). We hypothesized that LIMK1 competes with LIMK2 to bind β-catenin. LIMK2 interacts with β-catenin to stabilize β-catenin in the cytoplasm, whereas LIMK1 binds β-catenin to translocate it into nucleus. The subsequent WB and immunofluorescence assays confirmed our supposition. As shown in Fig. 5e and F, knocking down LIMK2, whereas overexpressing LIMK1 increased the maximum β-catenin level in the nucleus. Decreased LIMK2 accompanied by increased LIMK1 promotes CRC cell invasion and migration (Fig. 6a, b). See representative images in Supplementary figure S4B, S4C.

XAV939, a Wnt signaling pathway inhibitor, specifically inhibits β-catenin transcription. WB analysis showed that the β-catenin enhancement was inhibited when treated with 5 µM XAV939 (Fig. 6c). Nuclear accumulation of β-catenin caused by imbalanced LIMK1/LIMK2 was decreased with XAV939 treatment (Fig. 6d). Transwell assays validated that inhibition of the Wnt/β-catenin pathway impaired CRC cell invasion ability (Fig. 6e). These findings indicated that imbalanced expression of LIMK1 and LIMK2 occurred as CRC progressed and showed that LIMK1 and LIMK2 made a joint contribution to enhancing CRC cell metastasis via the Wnt/β-catenin pathway.

## Discussion

LIMK2, a member of the LIMK family, controls actin reorganization through phosphorylating and inactivating cofilin^17^. Previous studies have reported that LIMK2 was overexpressed in breast cancer^18^. Phosphorylated LIMK2 was also enhanced in human osteosarcoma^19^. However, in human CRC, Lourenco F.C. et al.^15^ confirmed that LIMK2 was decreased in CRC tissues compared with that in normal tissues. There were no previous reports about the relation between LIMK2 and precancerous lesions. SA is a type of precancerous lesion of CRC with a high cancer rate, and SA-related research has gradually gained attention as a new focus of CRC genesis^5^. Our research detected the expression of LIMK2 in normal, SA and CRC tissues and verified that LIMK2 was progressively downregulated with tumor development. LIMK2 might serve as a tumor marker to indicate the level of tumor progression.

Previous study of LIMK2 was focused on the role of cytoskeleton reorganization. LIMK2 controls actin cytoskeleton remodeling, silencing LIMK2 in osteoblasts increases their chemosensitivity^20^, the development of eyelid keratinocytes acquires LIMK2^21^, and LIMK2 is involved in cyclogenesis^22^. LIMK2 has also been proposed to function in nervous system development, and the studies were abundant^23–25^. Ko A.R. et al.^27^ reported that LIMK2 is involved in programmed neuronal necrosis^26^. As a direct target of p53, LIMK2 promotes pro-survival functions following DNA damage. LIMK2 has been reported as a potential therapeutic target for neurofibromatosis type 2^28^. However, LIMK2 showed different roles in malignant tumors. Overexpression of LIMK2 promoted aurora-A-kinase-mediated breast cancer^18^. In CRC, Lourenco F.C. et al. found that deletion of LIMK2 promoted CRC stem cell proliferation. So far, the mechanism of LIMK2 in CRC progression is still unknown. To further determine the mechanism of LIMK2 in CRC progression, we detected the expression of LIMK2 in CRC cell lines and utilized siRNA-transfected SW480 and HCT116 cells and plasmid-transfected LoVo and HT29 cells. Low serum of medium to culture cells was reliable to eliminate proliferation effect. Wound-healing assays and Transwell identified that LIMK2 silencing promotes CRC cell motility and invasiveness. Our data suggested that knocking down LIMK2 accelerated the G1–S transition in CRC cells, whereas overexpression of LIMK2 resulted in the opposite effect. These findings indicated that LIMK2 might serve as a tumor inhibitor in CRC.

EMT is a novel phenomenon correlated with tumor metastasis. Our research found that by knocking down LIMK2, a biomarker of epithelial cells (E-cadherin) was downregulated, whereas mesenchymal biomarker (vimentin) was upregulated. Interestingly, another epithelial biomarker, β-catenin, showed opposite changes, which indicated that β-catenin might serve other roles. β-cateninis a transcription factor that can translocate into the nucleus to activate the Wnt signaling pathway^29, 30^.

The Wnt signaling pathway comprises two pathways: the canonical Wnt signaling pathway and the non-canonical Wnt signaling pathway. β-catenin is a crucial regulator of canonical Wnt signaling via controlling a cluster of genes, including MMP-7, c-myc, Met, c-Jun, and so on. Previously, studies have identified the Wnt signaling pathway as a key signaling pathway involved in many processes of CRC^31^, such as CRC formation and metastasis. Our results showed that knocking down LIMK2 is associated with the accumulation of β-catenin in the nucleus, activating downstream target genes. These observations suggest that LIMK2 could activate the Wnt signaling pathway through controlling β-catenin nuclear translocation. According to literature, phosphorylation β-catenin at Ser675 promotes its dissociation from cell–cell contacts, induces its nuclear localization, and stimulates its transcriptional activity via Wnt pathways. We tested phosphorylation β-catenin Ser675 level of SW480 negative control (NC) and siLIMK2 cells by WB (Supplementary figure S5). Loss of LIMK2 enhanced p-β-catenin Ser675 and this may contribute to increase β-catenin level.

LIMK1, which shares a strong homology with LIMK2, promotes tumor proliferation and metastasis in various tumors, including CRC. LIMK1 enhances nuclear androgen receptor translocation, leading to prostate cancer cell proliferation and survival^32^. Research on zebrafish has confirmed that LIMK1 and LIMK2 lead to pancreatic cancer metastasis and angiogenesis^33^. In our previous study, we found a negative correlation between the expression of LIMK2 and LIMK1 in CRC^12^. The similar structure but different expression patterns of LIMK1 and LIMK2 in CRC suggest different roles in CRC development and progression. In the present study, we investigated the mechanism of LIMK1/LIMK2 in controlling CRC metastasis for the first time. Our data verified the correlation of LIMK2 and LIMK1 in CRC progression, suggesting that imbalanced expression of LIMK1 and LIMK2 contribute to CRC tumorigenesis. However, there was no obvious difference in Oxaliplatin sensitivity of silencing of LIMK1 and overexpression of LIMK2 CRC cells (Supplementary S6).

**Fig. 7:**
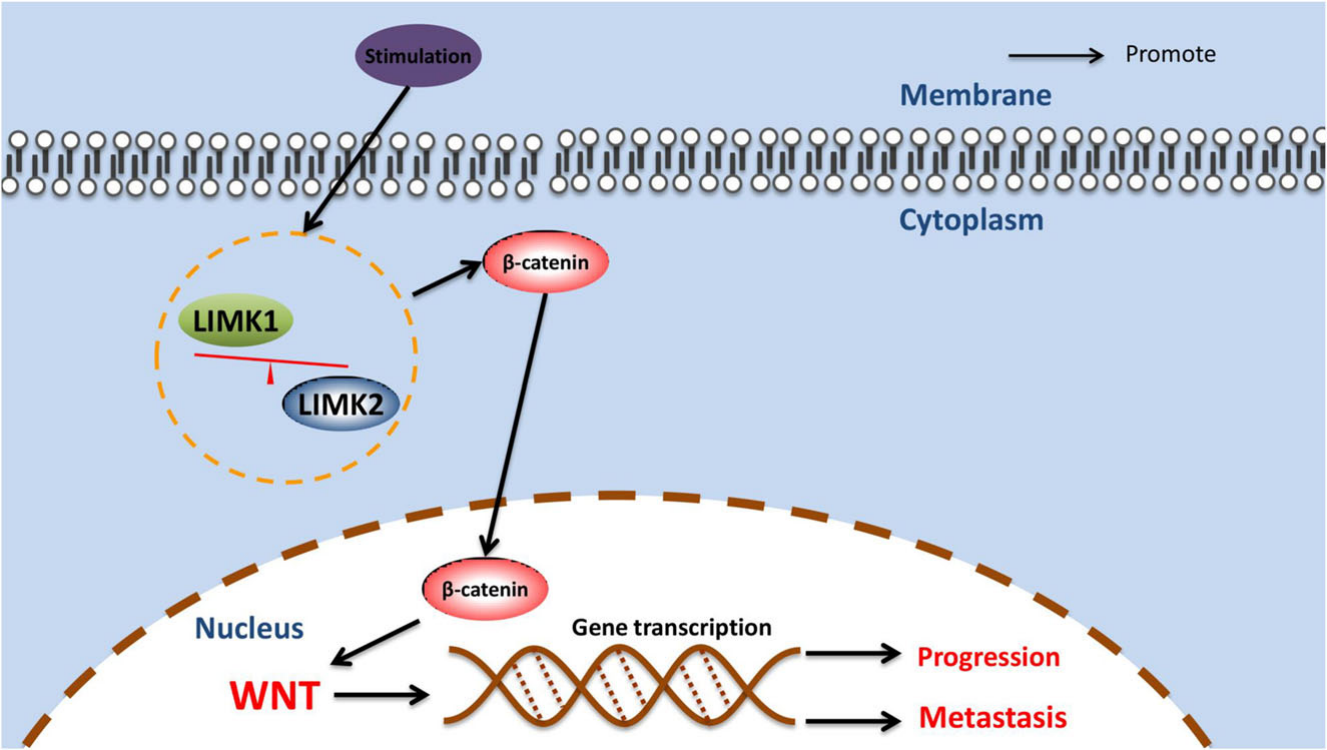
Hypothetical model showing that imbalanced LIMK1 and LIMK2 expression activates the WNT/β-catenin pathway and leads to CRC progression and EMT-induced metastasis

In summary, we observed progressive downregulation of LIMK2 from normal colon mucosa to SA tissue to CRC tissue, and LIMK2 was especially associated with tumor progression, suggesting its relation to tumor progression. Knocking down LIMK2 promoted CRC cell EMT-induced metastasis and accelerated the G1–S transition via activating the Wnt signaling pathway. Loss of LIMK2 and overexpression LIMK1 led to β-catenin concentration. The imbalanced expression of LIMK1 and LIMK2 could cooperate in the accumulation of β-catenin and thus the activation of a potential oncogenic process (Fig. 7). This study provides not only new insights into the molecular mechanism of tumor progression but also new clues for LIMK-based specific target drugs.

## Material and methods

### Cell line cultures

Human CRC cell lines (NCM460, SW480, SW620, HCT116, LS174t, HT29, and LoVo) were originally purchased from American Type Culture Collection (ATCC, Manassas, VA, USA). All cells were cultured in RPMI 1640 (HyClone, Logan, UT, USA) supplemented with 10% fetal bovine serum (Gibco-BRL, Invitrogen, Paisley, UK) and 1% penicillin/streptomycin (Invitrogen) at 37 °C with a humidity of 5% CO_2_.

### Tumor tissue samples

Human CRC tissues and corresponding normal colon tissues were obtained from patients with CRC who had undergone surgical resection. Human SA tissues and corresponding normal colon mucosa were obtained from patients who had undergone endoscopic mucosal resection or endoscopic submucosal dissection. All patients were from Nanfang Hospital, First Affiliated Hospital of Southern Medical University, Guangzhou, China. The study was approved by the Ethics Committee of Southern Medical University and all aspects of the study comply with the Declaration of Helsinki.

### IHC

See supplementary materials and methods for details.

### Western blotting analysis

WB was performed as previously described^34^, using monoclonal anti-LIMK2 (1:1000; Abcam, Cambridge, MA, USA) and polyclonal anti-LIMK2 (1:1000; Proteintech, Chicago, IL, USA), anti-E-cadherin, anti-Vimentin, anti-Met, anti-c-myc, anti-c-jun (1:1000; Cell Signaling Technology, Danvers, MA, USA), anti-LIMK1, anti-β-catenin, anti-lamin B (1:1000; Proteintech, Chicago, IL, USA). The loading control was a mouse anti-GAPDH monoclonal antibody (1:10000; Proteintech, Chicago, IL, USA).

### RNA isolation, reverse transcription, and quantitative real-time PCR

Total RNA was extracted using Trizol reagent (Invitrogen). To quantify LIMK2 and LIMK1 expression, total RNA was polyadenylated and underwent reverse transcription using PrimeScript RT Master Mix (Takara). Quantitative real-time PCR (qRT-PCR) was performed in triplicate using TB Green™ Premex Ex Taq™ II (Tli RNaseH Plus; Takara) and a Roche Light Cycler 480 Real-Time PCR System (Roche Diagnostics, Mannheim, Germany). Relative gene expression levels were calculated by using the 2-ΔΔCt method. The ΔCt value of each sample was calculated using GAPDH as an endogenous control gene. See the primers in the Supplementary materials and methods for details.

### siRNA-mediated gene silencing

siRNAs were transfected at a working concentration of 100 nmol/L using Lipofectamine 3000 reagent (Invitrogen; Carlsbad, CA, USA). Transfection of control siRNA and LIMK2 siRNA (GenePharma, Shanghai, China) in SW480 and HCT116 cells according to the manufacturer’s instructions. See the siRNA sequences in the Supplementary materials and methods for details. Proteins were extracted from sub-confluent cells during the exponential phase of growth.

### Plasmid constructs

Flag-LIMK2 and Flag-control were constructed in GV141 cells. Tagged Flag-LIMK2 was generated by standard PCR techniques (Shanghai GenePharma Co., China). The sequences used to generate the recombinant vectors were as follows: LIMK2 sequence (ACAACTGCCTCATCAAGTTG) and control sequence (TTATTAGGAAAGGACAGTGGG). The coding sequences for all Flag-LIMK2 and Flag-control constructs were verified by sequencing analysis. The HA-LIMK1 plasmid was a gift from the Department of Pathology of Nanfang Hospital of Southern Medical University.

### Preparation of lentiviral vectors

The LIMK2 shRNA sequence was sense 5′-GGATGCACATCAGTCCCAA-3′ and the control shRNA sequence was sense 5′-TTCTCCGAACGTGTCACGT-3′. The human LIMK2 construct was generated by cloning the PCR-amplified full-length human LIMK2 cDNA with the transcript NM_001031801. The shRNA lentivirus and the control lentivirus (Obio Technology, Shanghai, China) were transfected into SW480 cells according to the manufacturer’s instructions.

### Cell proliferation assay

See supplementary materials and methods for details.

### Cell cycle analysis

See supplementary materials and methods for details.

### Cell migration analysis

See supplementary materials and methods for details.

### Wound-healing assay

See supplementary materials and methods for details.

### Immunofluorescence

CRC cells were fixed in 4% paraformaldehyde and permeabilized in 0.5% Triton at 37 °C for 30 min. In Fig. 5d, LIMK2-Flag and LIMK1-HA were conducted into LoVo. LoVo were incubated overnight with the anti-Flag and anti-HA (Proteintech, Chicago, IL, USA) antibodies. In Fig. 5f, the cells were incubated overnight with the anti-LIMK2 (Abcam) and anti-β-catenin (Proteintech, Chicago, IL, USA) antibodies. Cells were then incubated at 37 °C with Alexa Fluor® 594-conjugated goat anti-mouse IgG (H + L) (Zhongshan Biotech, Beijing, China) and Alexa Fluor® 488-conjugated goat anti-rabbit IgG (H + L) (Zhongshan Biotech, Beijing, China). Then, the cells were stained with DAPI. Images were captured under Confocal Laser Scanning Microscope Olympus-FV1200 (Olympus, Japan). The experiments were performed at least three times.

### Co-immunoprecipitation

See supplementary materials and methods for details.

### Animals and the tumor growth assay

Male BALB/c nude mice aged 4–5 weeks were purchased from the Laboratory Animal Services Centre of Guangdong Province. Animal handling and experimental procedures were approved by the Animal Experimental Ethics Committee of Southern Medical University. For the tumor growth assay, 1 × 10^6^ SW480/shLIMK2 cells and SW480/shLIMK2 NC cells were independently injected subcutaneously into the left back of nude mice (*n* = 6/group). The tumor volume was calculated using the following formula: *V* = 0.5 × *D* × *d*2, where *V* represents volume, *D* represents the longitudinal diameter, and *d* represents the latitudinal diameter.

### Tumor metastasis assay

To determine the liver metastatic potential of the cancer cells in vivo, we injected 5 × 10^6^ SW480/shLIMK2 cells and SW480/shLIMK2 NC cells into nude mice (*n* = 6/group) through the spleen. The mice were all killed 2 months later, at which time the individual organs were removed, and the metastatic tissue was analyzed using hematoxylin and eosin staining.

### Statistical analysis

Data were analyzed using SPSS version 17.0 software (SPSS; Chicago, IL, USA). IHC score was analyzed by Kruskal Wallis. Student’s *t* tests and one-way analysis of variance tests were carried out for the qRT-PCR data and to calculate the tumor growth curves. The correlation between LIMK2 and LIMK1 was determined using Spearman’s rank correlation test. Data are presented as the mean ± SEM, and *P* values<0.05 were considered statistically significant.

## Electronic supplementary material

Supplementary Figure Legends

Supplementary Table

Supplementary FigureS1

Supplementary FigureS2

Supplementary FigureS2E

Supplementary FigureS3A

Supplementary FigureS3B

Supplementary FigureS4 S5

Supplementary FigureS6

Supplementary FigureS7

Supplementary materials and methods
