# Supplementary material for: Imbalanced LIMK1 and LIMK2 expression leads to human colorectal cancer progression and metastasis via promoting β-catenin nuclear translocation
Source: Cell Death Dis. 2018 Jul 3;9(7):749. doi: 10.1038/s41419-018-0766-8 (PMC6030168; doi:10.1038/s41419-018-0766-8)
Supplement: Supplementary file 1 — Supplementary Figure Legends [file 41419_2018_766_MOESM1_ESM.docx]

**Figure S1.** **LIMK2 is down-regulated in human CRC tissues. (A)**Western blot analysis of LIMK2 in 10 paired tumor tissues (T) and adjacent non-tumor tissues (N). Representative photographs are shown.

**Figure S2**. **Reduced LIMK2 promotes metastasis ability in vitro.**

(A)HCT116 cells were transiently transfected with siLIMK2 and siRNA NC. HT29 cells were transiently transfected with LIMK2 and NC vectors. Western blot analysis was performed to detect the expression of LIMK2. (B and C) The invading cells of the transwell assay were counted in HCT116 cells with siLIMK2 and HT29 cells with plasmid LIMK2. Bars represent the number of invaded cells. Error bars represent mean±S.D. from three independent experiments. ***P<0.001. **P<0.01.(D)Wound healing assays were used for detecting the motility ability in HCT116 cells with siLIMK2. Bars represent migration index of treated or control cells. The distance migrated by treated cells was relative to that migrated by control cells. The asterisk (*) indicates P<0.05. (E) Representative figures of wound healing assays of SW480, LoVo, HCT116 cells were shown.

**Figure S3**. **Reduced LIMK2 accelerates cell cycle progression and proliferation of CRC cells.** (A) The cell cycle phases of treated cells were evaluated by PI staining flow cytometry after transfection for 48h. Data was expressed as means±SD (n=3) (* P<0.05, **P<0.01). The cell cycle protein was assessed by Western Blot. (B) CCK8 was used to assess LIMK2 on cell proliferation. Representative images from three independent experiments. (* p<0.05).

**FigureS4. Validation of endogenous interaction between LIMK1 and β-catenin.**(A) Co-immunoprecipitation revealed the relationship between β-catenin

and LIMK1 in non-transfected SW480 cells.

**FigureS5.** **Imbalanced LIMK2 and LIMK1 promotes CRC cell invasion and migration.** (A) The wound healing assay was conducted in SW480 and SW480 transfected with LIMK1, co-transfected with LIMK2 or siLIMK2, respectively. Representative images from three independent experiments.

(B) The invading cells of the transwell assay were counted in SW480 and SW480 transfected with LIMK1, co-transfected with LIMK2 or siLIMK2, respectively. Representative images from three independent experiments.

**FigureS6.** **Decreased LIMK2 enhances phosphorylation β-Catenin at Ser675.** (A) Phosphorylation β-Catenin Ser675 level of SW480 NC and siLIMK2 cells was detected by Western Blot.

**FigureS7.** **Silencing LIMK1 and overexpression of LIMK2 had no influence on sensitizing CRC cells towards Oxaliplatin.** (A) CCK8 assays was used to evaluate the proliferative ability of silencing LIMK1 and overexpression of LIMK2 in SW480 cells towards Oxaliplatin (10ug/ml) for 24h. Representative images from three independent experiments.
