# Supplementary material for: Imbalanced LIMK1 and LIMK2 expression leads to human colorectal cancer progression and metastasis via promoting β-catenin nuclear translocation
Source: Cell Death Dis. 2018 Jul 3;9(7):749. doi: 10.1038/s41419-018-0766-8 (PMC6030168; doi:10.1038/s41419-018-0766-8)
Supplement: Supplementary file 2 — Supplementary Table [file 41419_2018_766_MOESM2_ESM.docx]

**IHC Score of Normal,SA and Colorectal Cancer**

| Normal | Cancer | SA |
| --- | --- | --- |
| 3 | 0 | 0 |
| 3 | 1 | 0 |
| 3 | 2 | 2 |
| 2.67 | 2 | 0.67 |
| 2.33 | 0.33 | 1 |
| 3 | 1 | 2 |
| 3 | 0.33 | 1.67 |
| 2 | 0.33 | 3 |
| 3 | 1 | 2 |
| 3 | 1 | 1.67 |
| 2 | 0.67 | 2 |
| 1.67 | 1 | 2 |
| 2 | 0 | 2 |
| 1.67 | 0 | 0.67 |
| 0.67 | 0.33 | 1 |
| 3 | 0.33 | 0.67 |
| 3 | 0 | 0.33 |
| 0.67 | 0.33 |  |
| 2 | 0 |  |
| 3 | 0 |  |
| 2.67 | 0 |  |
| 2 | 0 |  |
| 3 | 0 |  |
| 3 | 1 |  |
| 2 | 0 |  |
| 2 | 0 |  |
| 3 | 0 |  |
| 3 | 1 |  |
| 2.67 | 1.67 |  |
| 1 | 1 |  |
| 3 | 1 |  |
| 3 | 0 |  |
| 2.67 | 0 |  |
| 1.67 | 0 |  |
| 3 | 1 |  |
| 1.67 | 0 |  |
| 3 | 0 |  |
| 0.67 | 0.33 |  |
| 3 | 0.33 |  |
| 1.67 | 0 |  |
| 1.67 | 0 |  |
| 2 | 0 |  |
