# Supplementary figures and images for: Imbalanced LIMK1 and LIMK2 expression leads to human colorectal cancer progression and metastasis via promoting β-catenin nuclear translocation
Source: Cell Death Dis. 2018 Jul 3;9(7):749. doi: 10.1038/s41419-018-0766-8 (PMC6030168; doi:10.1038/s41419-018-0766-8)

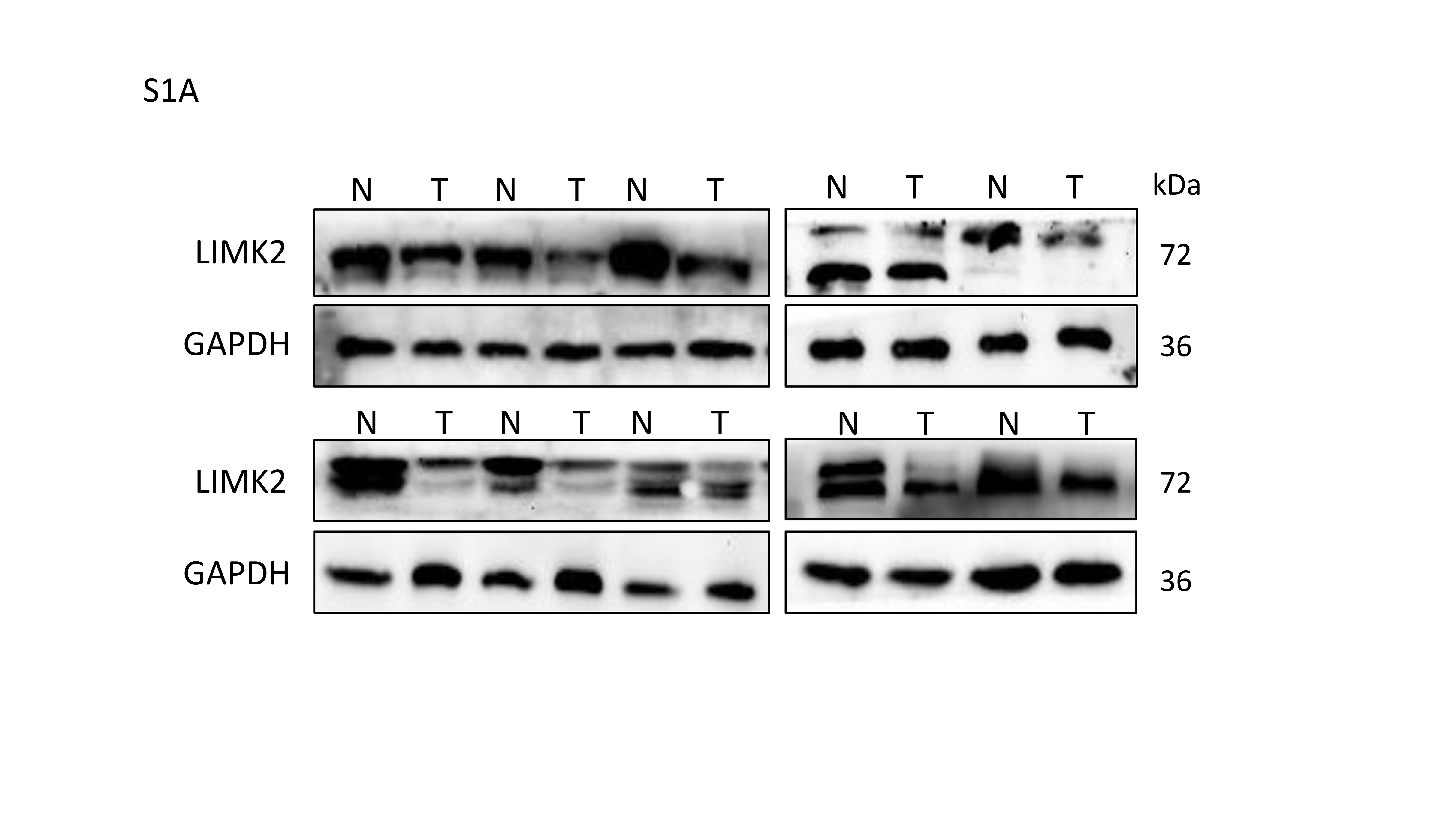

Supplement: Supplementary file 3 — Supplementary FigureS1 [file 41419_2018_766_MOESM3_ESM.jpg]

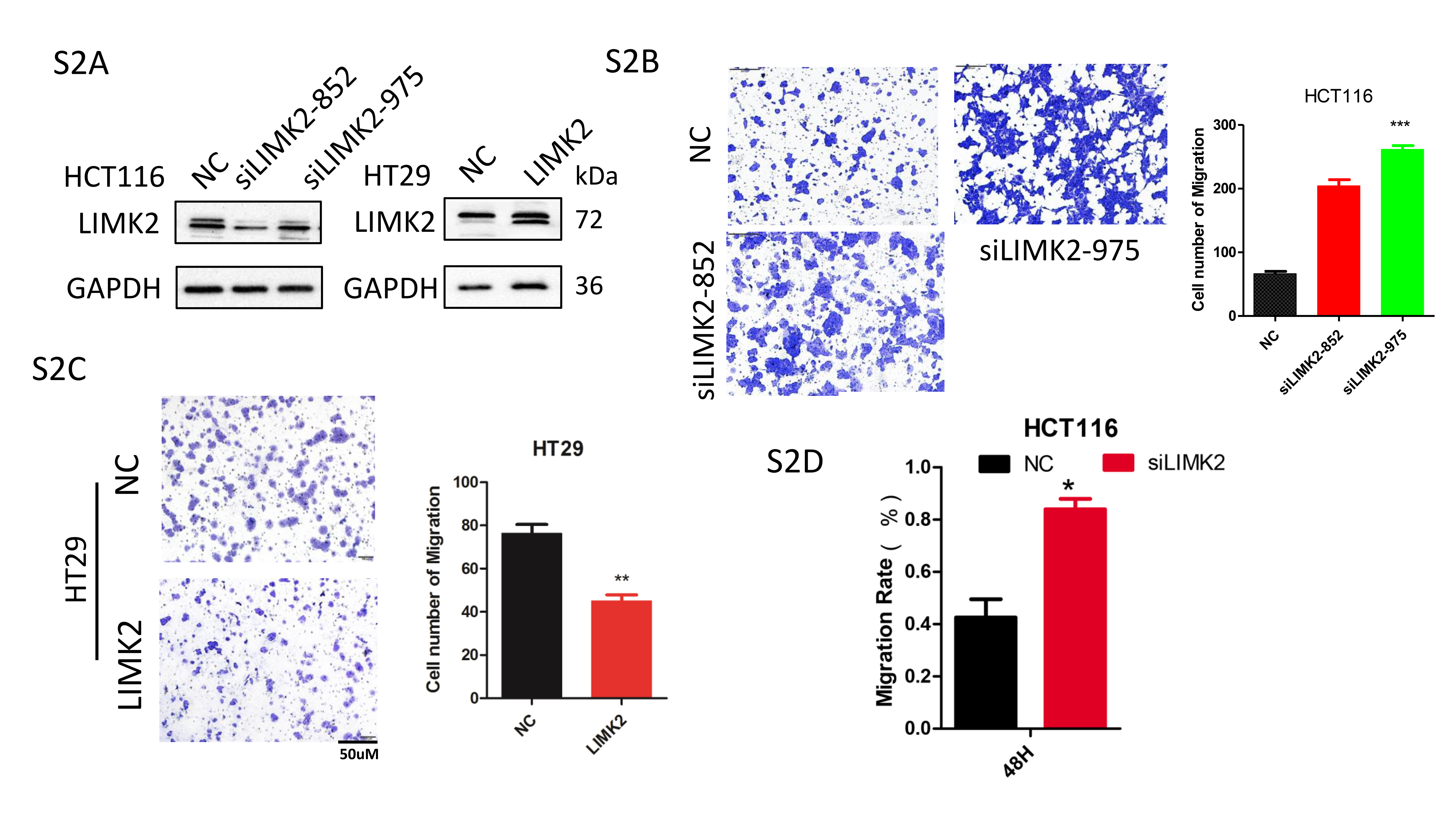

Supplement: Supplementary file 4 — Supplementary FigureS2 [file 41419_2018_766_MOESM4_ESM.jpg]

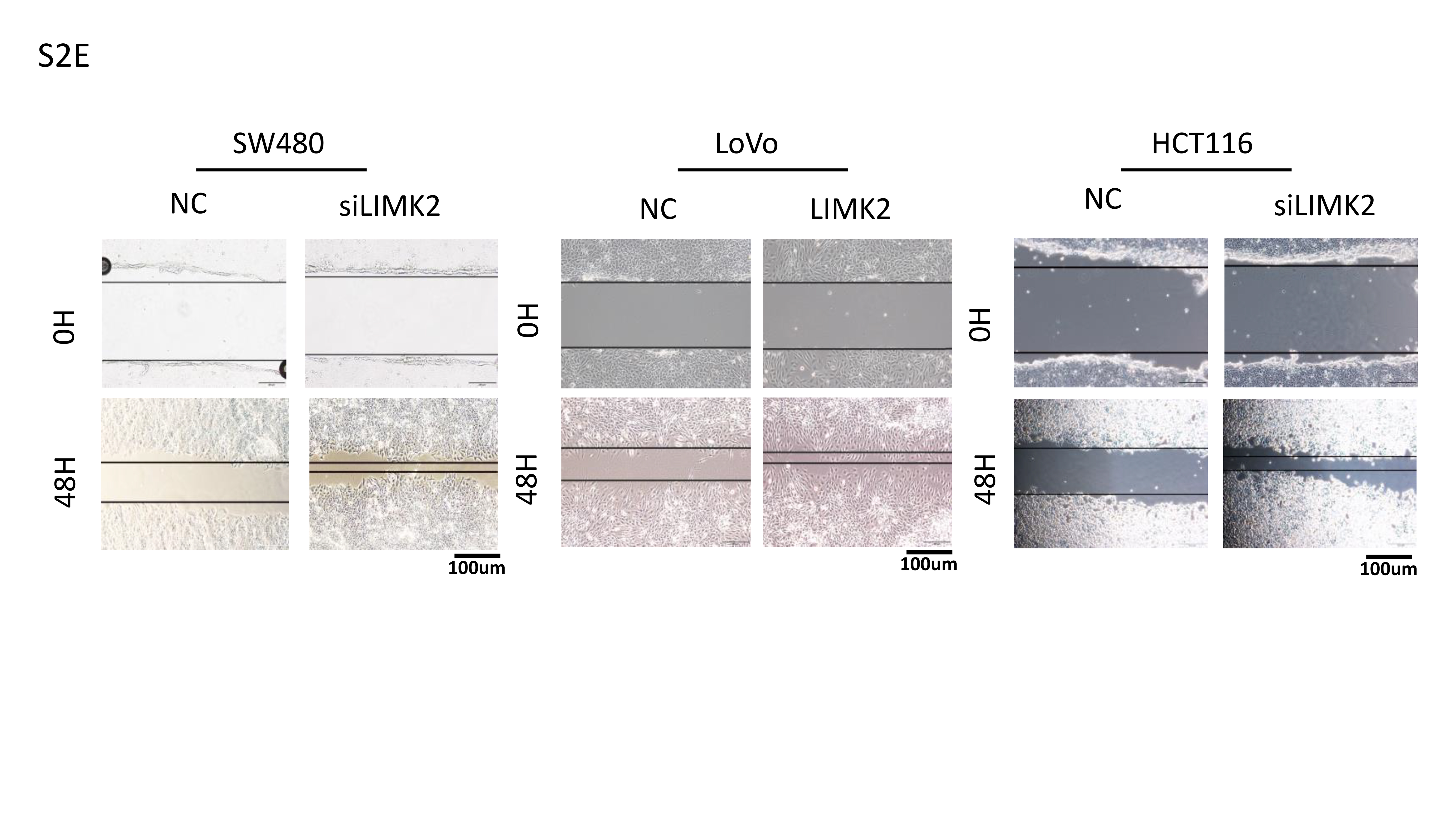

Supplement: Supplementary file 5 — Supplementary FigureS2E [file 41419_2018_766_MOESM5_ESM.jpg]

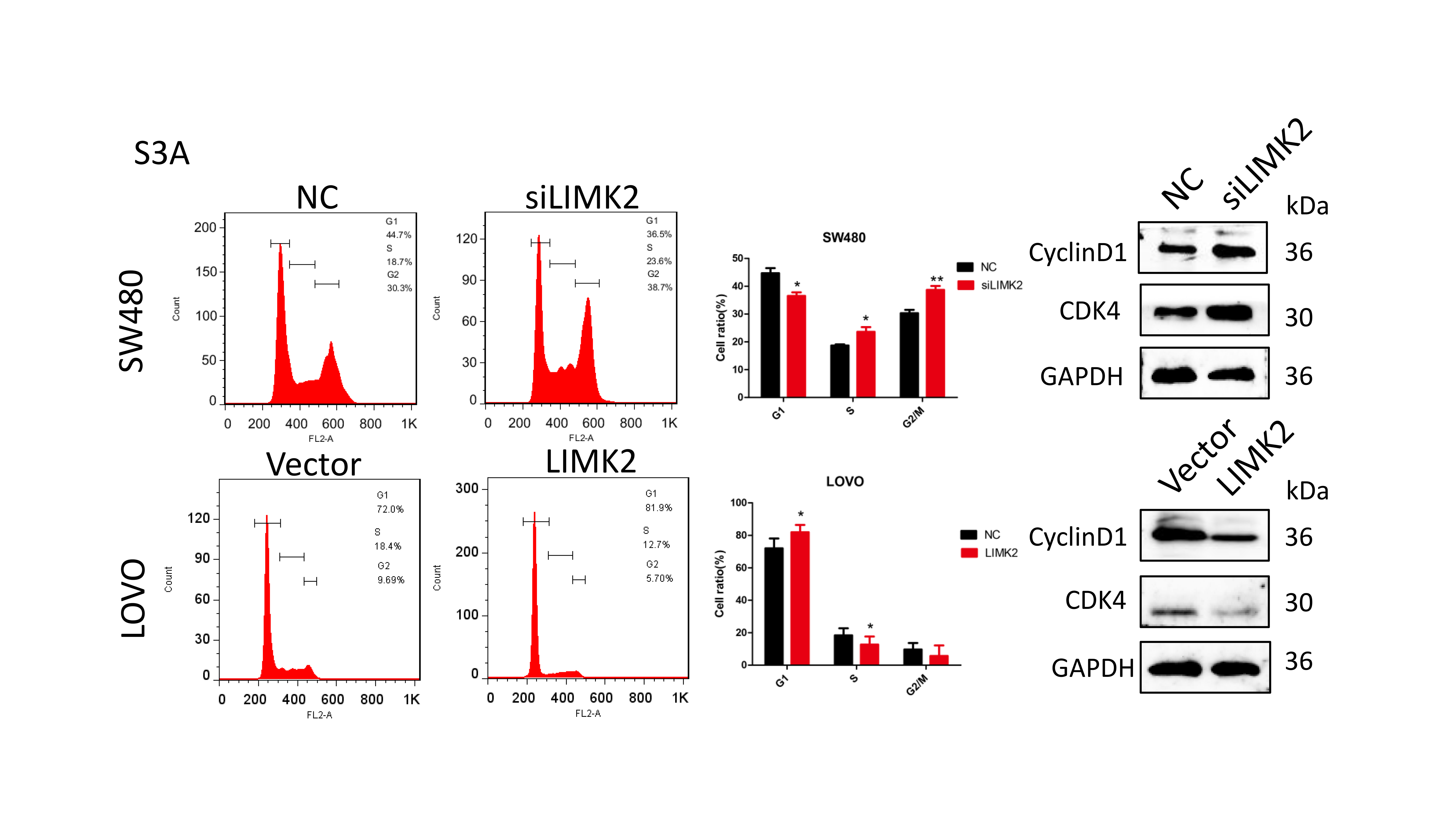

Supplement: Supplementary file 6 — Supplementary FigureS3A [file 41419_2018_766_MOESM6_ESM.jpg]

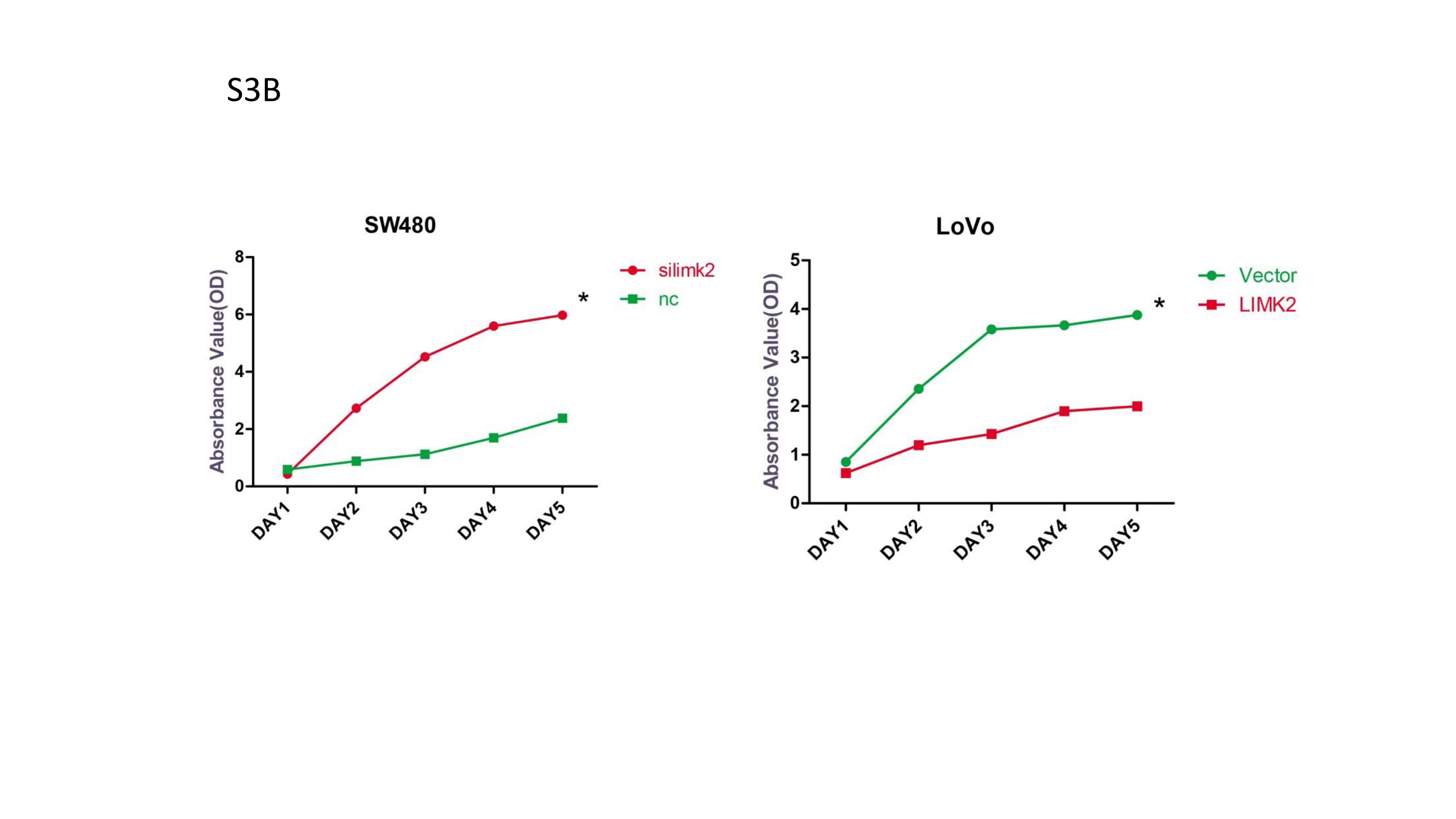

Supplement: Supplementary file 7 — Supplementary FigureS3B [file 41419_2018_766_MOESM7_ESM.jpg]

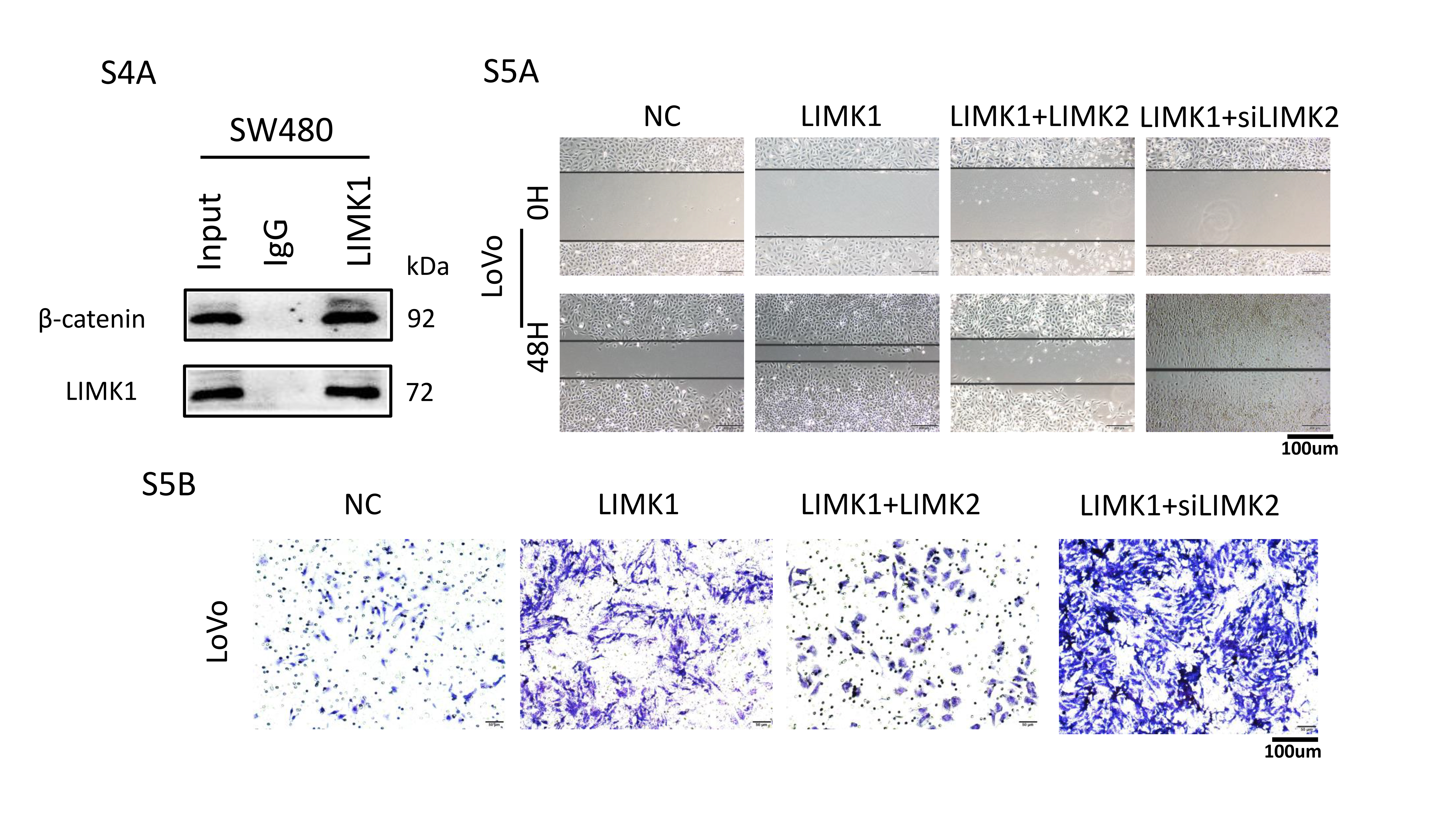

Supplement: Supplementary file 8 — Supplementary FigureS4 S5 [file 41419_2018_766_MOESM8_ESM.jpg]

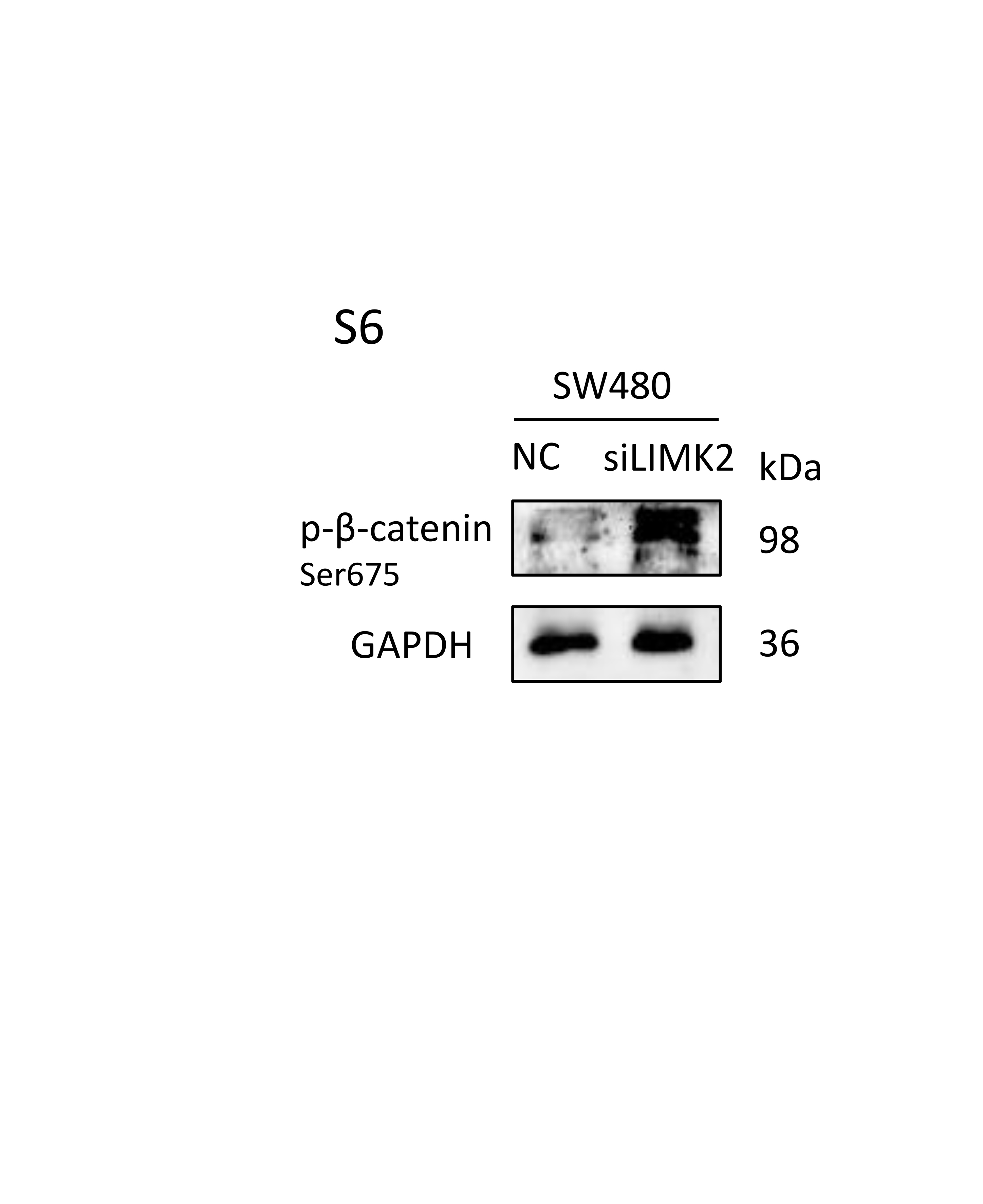

Supplement: Supplementary file 9 — Supplementary FigureS6 [file 41419_2018_766_MOESM9_ESM.jpg]

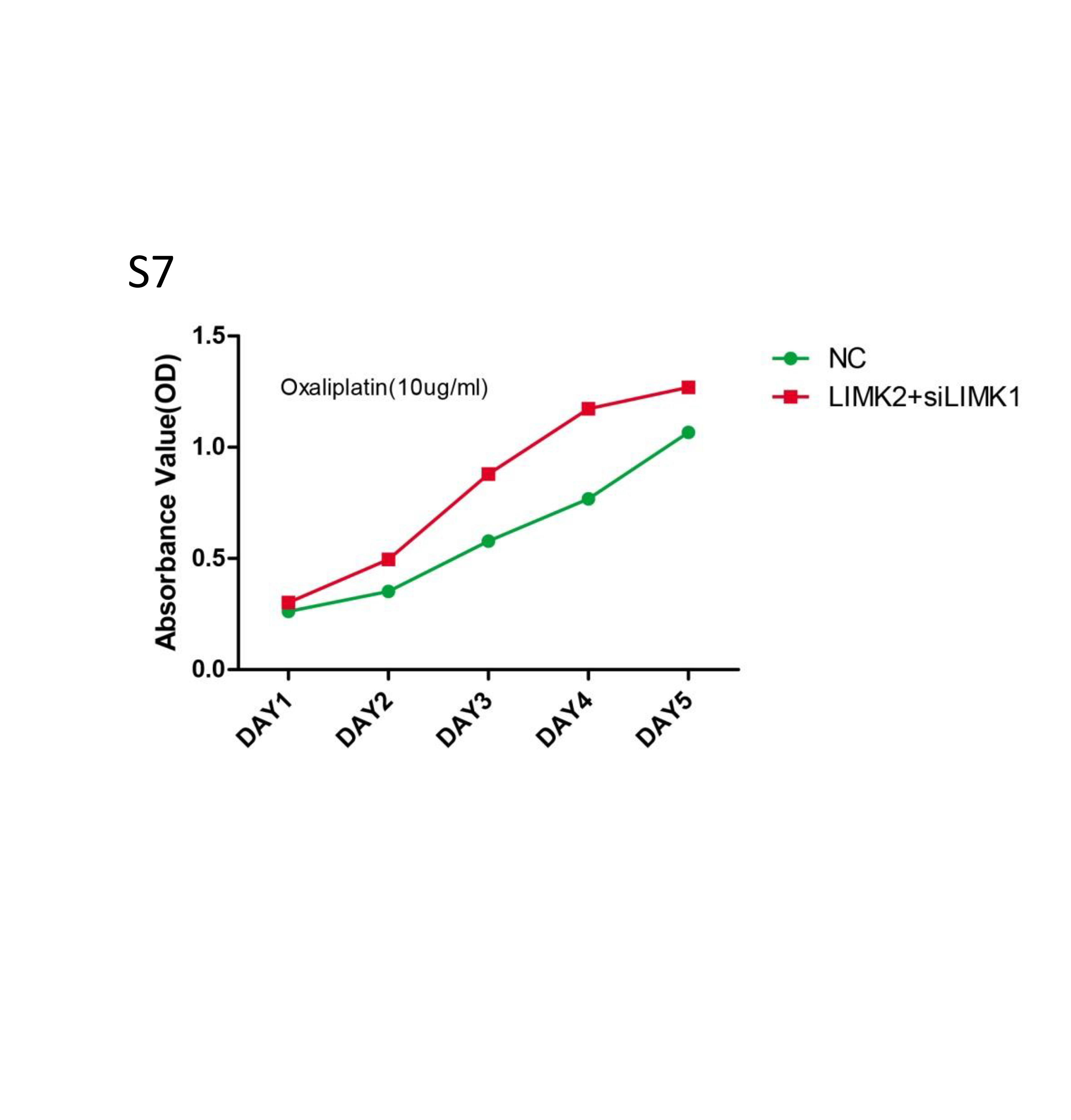

Supplement: Supplementary file 10 — Supplementary FigureS7 [file 41419_2018_766_MOESM10_ESM.jpg]
