# Supplementary material for: Imbalanced LIMK1 and LIMK2 expression leads to human colorectal cancer progression and metastasis via promoting β-catenin nuclear translocation
Source: Cell Death Dis. 2018 Jul 3;9(7):749. doi: 10.1038/s41419-018-0766-8 (PMC6030168; doi:10.1038/s41419-018-0766-8)
Supplement: Supplementary file 11 — Supplementary materials and methods [file 41419_2018_766_MOESM11_ESM.docx]

**Supplementary Materials and Methods**

**Immunochemistry.**

Paraffin-embedded tissue blocks were cut into 5 μm sections and transferred to glass slides. Then the section weredeparaffinized with xylenes and rehydrated. After treatment with 3% hydrogen peroxide in methanol to quench the endogenous peroxidase activity, the section were submerged into citrate buffer and high-pressure boiled for antigenic retrieval, followed by incubation with 1% bovine serum albumin to block the nonspecific binding. Rabbit anti-LIMK2 (1 : 200; Abcam, Cambridge, MA, USA) or rabbit anti-LIMK1(1 : 200; Proteintech, Chicago, IL, USA) was incubated with the section overnight at 4 °C. For negative controls, the rabbit anti-LIMK2 or anti-LIMK1antibody was replaced with normal goat serum. After washing, the slides were treated with anti-rabbit secondary antibody (Zhongshan Biotech, Beijing,China). The tissue section were incubated with 3,3-diaminobenzidin and counterstained with hematoxylin, dehydrated and mounted. The section were

reviewed and scored independently by two observers, based on both the proportion

of positively stained tumor cells and the intensity of staining. The intensity of staining was graded according to the following criteria: 0 (no staining); 1 (weak staining= light yellow), 2 (moderate staining= yellow brown), and 3 (strong staining= brown).The y-axis indicated IHC score. The staining was rated by three pathologists, respectively. And the average score was shown in Supplementary Table. An optimal cutoff value was identified: the score of ≥2 was used to define tumors as high LIMK2 expression and<2 as low expression of LIMK2. The tissues from the BALB/c nude mice were stained as the same with Rabbit anti-KI67 (1 : 200; Abcam, Cambridge, MA, USA).

**Cell proliferation assay**

### The siRNA- or plasmid-transfected cell lines were seeded on 96-well plates at an initial density of 1-2 × 103/well. At each time point, cells were detected by a Cell Counting Kit-8 Kit (Dojindo; Kumamoto, Japan) following the kit’s assay protocol. Cell counting kit-8 assay. Each group of cells (1 × 10^3^ cells/well) was seeded onto 96-well plates and incubated overnight. 10 µl of the Cell Counting Kit-8 reagent was added to each well, and the cells were incubated for 2 h at 37 °C. Finally, the spectrophotometric absorbance of each sample was measured using a microplate reader (Synergy HT, Bio-Tek) at 450 nm. Each group of cells was analyzed using five replicates, and the experiments were performed in triplicate.

**Cell cycle analysis.**

The effect of LIMK2 on cell cycle progression was determined by flow cytometry using a Cell Cycle Detection Kit (KeyGEN BioTECH,Nanjing, China). The transfected cells were seeded in six-well plates and cultured for 2 days. 2 × 10^5^ cells were then collected and fixed with cold 70%ethanol for 24 h at 4 °C. Next, cells were incubated with 0.5 mg/ml of propidium iodide along with 0.1 mg/ml of RNase A. Cell cycle analysis was performed using flow cytometry (BD, San Diego, CA, USA).

**Cell migration analysis.**

Cells from the serum-free medium (1 × 10^5^ cells/100 μL) were added to the top chamber of each 8-mm–pore transwell chamber (Corning Star; Cambridge, Mass, USA). The bottom chamber was prepared using 10% FBS as a chemoattractant. Cells were allowed to migrate through the porous membrane for 20 h at 37℃. The cells that stuck to the lower surface of the membrane were treated with a ﬁxation/staining solution (0.1% crystal violet, 1% formalin, and 20% ethanol) for visualization. The cells were counted under a microscope in 5 randomly selected ﬁelds (original magniﬁcation, ×200). At least 4 chambers from 3 different experiments were analyzed.

**Wound healing assay.**

SW480 or LoVo cells were seeded into 6-well tissue culture plates at a density that after 24 h of growth, they should reach ~70-80% confluence as a monolayer. The monolayer were scratched gently and slowly with a new 10ul pipette tip across the center of the well. While scratching across the surface of the well, the long-axial of the tip was perpendicular to the bottom of the well. Wells were washed gently twice with PBS to remove the detached cells and replenished with DMEM with 0.5%FBS. Photos were taken at 12hour, 24 hours,36 hours ,and 48hours,respectively. The gap distance was quantitatively evaluated using Photoshop. Each well was documented for three times , and each experimental group were repeated for three times.

**Co-immunoprecipitation.** Co-immunoprecipitations of endogenously

expressed proteins were performed in SW480 cells. In all, 5 × 10^6^ cells were

lysed using ice-cold RIPA buffer containing protease and phosphatase inhibitors.

After a 30-min incubation at 4 °C, total extracts were clarified by centrifugation at

12 000 r.p.m. for 30 min.

In IP, the cell extracts were precleared with 50 μl protein A+G agarose (Yeasen, Shanghai, China) for an hour at 4 °C, and the cleared extracts were immunoprecipitated with 2 μg of the anti-Rabbit IgG and anti-β-catenin ,respectively. The immune complexes were washed three times with wash buffer for 3 min each time to avoid unspecific binding of associated proteins. Every 30ml wash buffer concluded 3ml Tris-HCl(pH 7.6), 3ml NaCl, 3ml EDTANa2, 3ml Glycerol and 18ml PBS. After the third wash, immunoprecipitates were re-suspended in SDS-PAGE sample buffer containing loading dye and used for western blotting. In WB detection , we used anti-LIMK2 (1:1000; Proteintech, Chicago, IL, USA) , anti-β-Catenin (1:1000; Proteintech, Chicago, IL, USA).
